# Teledermatology to Support Self-Care in Chronic Spontaneous Urticaria

**DOI:** 10.2196/81830

**Published:** 2025-11-13

**Authors:** Laura Schuehlein, Martin Peters, Graham Jones

**Affiliations:** 1 Novartis AG, Basel, Switzerland; 2 Tufts University Medical Center, 800 Washington St, Boston, MA, 02115, United States, 1 8572757045

**Keywords:** dermal imaging, chronic spontaneous urticaria, diagnosis, teledermatology, self care

## Abstract

Chronic spontaneous urticaria (CSU) is an autoimmune prompted skin disorder, whose hallmarks include the unpredictable onset of hives and itch. Symptom duration typically exceed 6 weeks, and flares can occur for up to 5 years or longer if untreated, impacting potentially any area of the body. The absence of obvious triggers and the variation in onset frequency often delays formal diagnosis which on average is approximately 2 years from first presentation. Initial standard of care is the use of low through to higher strength antihistamines in the first instance, with eventual escalation to prescription anti-inflammatory agents and potentially biologics once patients are under managed care. The societal impacts of delays in diagnosis are marked, with data suggesting CSU impacts up to 1% of the population, primarily of working age and with twice the prevalence in women. Herein, we advocate for the deployment of smartphone imaging and generative artificial intelligence technology to improve detection and early management of CSU through integrated self-care approaches. Such approaches embodying the tenets of P4 personalized medicine could have sustained impact on the disease through awareness campaigns, reducing the burden on the dermatology community and facilitating earlier access to curative therapeutic interventions.

## Introduction

Dermatological conditions are estimated to afflict nearly 2 billion people globally, yet because of historical shortages of expert dermatologists, the majority of cases are addressed by general practitioners, resulting in lowered diagnostic accuracy and delays in patients receiving optimized care. Chronic spontaneous urticaria (CSU) is representative of this conundrum, with patients typically addressing symptoms periodically and episodically with antihistamine agents, which provide short-term relief from symptoms but do not address the root cause. With powerful disease modifying prescription agents now available, it becomes imperative that patients’ symptomatic presentations are documented, then adequately assessed to allow escalation as soon as practicable [1-6]. Given the rich structural and topological elements associated with symptoms (eg, hives, itch), there is natural interest in the use of imaging technologies coupled with computational tools to improve diagnosis [7]. In one recent teledermatological study involving over 16,000 cases, a deep learning system was deployed on photographic images and demonstrated high diagnostic accuracy [8]. The rapid evolution of mobile health technologies has now resulted in standardized methods for sharing of personal health images using smartphone technologies and has resulted in dedicated support apps for CSU such as CRUSE [9]. A key to the long-term deployment of and adherence to these solutions lies at the intersect of behavioral and social sciences [10], and we explore tactics herein which may lead to more widespread adoption.

## Discussion

**Figure 1.**
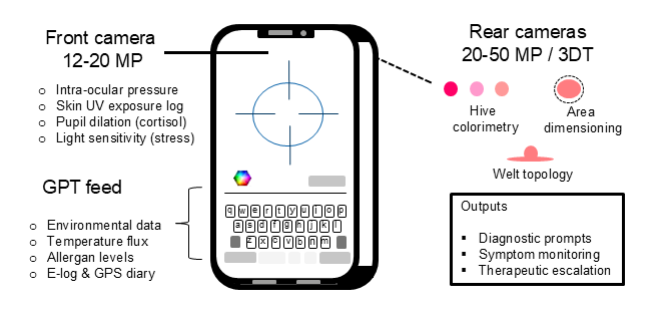
Specimen CSU relevant passive and active monitoring. CSU: chronic spontaneous urticaria.

The impact of digital health on modern medicine is being witnessed in multiple areas, most dramatically, where patients obtain medical-related information on symptoms and potential remedies [11]. Though quality and validity of this information varies greatly, there is increased awareness surrounding skin-related diseases together with methods, typically using smartphone cameras, to assess visual characteristics to aid diagnosis [12]. In the case of CSU, some of the visible hallmarks are hives and welts whose appearance, locations, dimensions, and topology are potentially useful indicators in diagnosis, self-care, and outcome assessments (Figure 1) [4].

Although some way-off from providing clinical grade characterization, these features could be useful in (1) raising awareness and prompting a patient to seek medical care, (2) serving as time-stamped reference points to track disease progression, and (3) confirming any observed treatment effects, for example, from topical adjuvants, over the counter symptomatic agents or prescribed disease modifying therapies. If used with precision and guidance, such information could become a key component in the patient’s electronic health record, similar to how patient-recorded outcomes or self-assessment questionnaires have utility as part of a patient’s composite case history. Achieving desired medical grade standard will require active dialog with professional dermatological societies, including standardized formats, rules and metrics for image capture and storage, procedures to validate images against controls, verification and authentication that it is associated with the patient, and informed consenting for upload and sharing of data [13]. Nonetheless, rapid progress is being observed and there is every reason to believe that such approaches will be embraced by the medical community and become integral components of telehealth based care of dermatological conditions in the future.

While these efforts progress, there is need to consider additional aspects of self-care which can help improve adherence to best practices and promote lifelong engagement with patients beyond addressing visual symptoms. A key to this approach is to consider behavioral and environmental factors which impact the patient on a perpetual basis, to drive engagement at multiple touch points. Ideally these can be achieved using a single device, and it turns out that the modern smartphone offers multiple options in this regard. For example, staple environmental factors including allergen levels in the atmosphere, ambient temperature, barometric pressure and relative humidity are all potential contributing factors in triggering immune responses [14]. Precision data, linked to GPS coordinates, can be provided on a real-time basis to smartphones, and artificial intelligence (AI or GPT based alerts could be directed to the patient—eg, on entering an area with high levels of specific pollens. This could be of additional relevance if recent dermatological images of CSU-related features (Figure 1) are stored on the smartphone and used to prompt alerts, for example, need for symptomatic therapy dose escalation. There may also be potential for pre-emptive action to avoid disease triggering. The environmental-based data could alert a patient to reconsider an action (eg, walking in a particular area) but the front camera of the smartphone could also be of utility. The sensors of this camera could be engaged automatically (eg, unlocking via retina scan) and have the ability to measure numerous features including intraocular pressure [15] and pupil dilation [16]. Both metrics correlate with elevated levels of plasma cortisol, which itself is a biomarker for stress and a potential trigger for CSU [17]. Likewise, light sensitivity and heightened UV exposure to facial skin are also potential immune-mediated triggers and could be detected and measured through the front camera sensor (Figure 1) [18]. Taken in sum, there are multiple opportunities to frame CSU patient self-management through the medium of the smartphone and in doing so, provide an effective mechanism for risk avoidance, real-time education, and longitudinal disease monitoring.

### Integrating Into Managed Care

**Figure 2.**
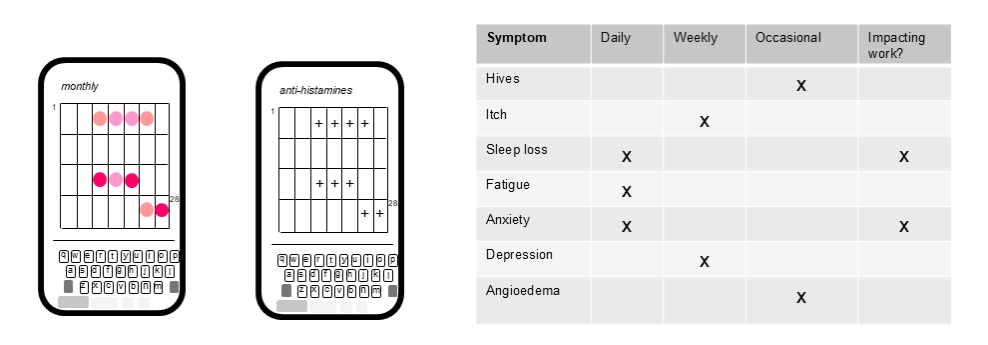
Combining images and medication log with activities of daily living data to assess control.

Successfully implementation of a smartphone-based tracking tool into managed care for CSU patients will require careful consideration of the patient-provider interface. Whether managed by a specialist dermatologist or primary care physician, there are a number of common scoring tools available including the Urticaria Activity Score (UAS), Urticaria Control Test (UCT), and Chronic Urticaria Quality of Life Questionnaire (CU-Q2oL) [19]. These scales can provide key insights to disease history and impact and inform treatment plans. This said, their deployment is not widespread or uniform, as there is a natural tendency for providers to treat presented visual symptoms, initially with antihistamines. Since a high percentage of patients are refractory to antihistamine treatment administered at the standard dose, there is potential for patients to disengage from care, resulting in impact to quality of life. The envisioned app could provide, on demand, a digital log of any visual symptoms, a patient provided log of impact on quality of life, and potentially a log of medication history from which to assess impact of both therapeutics themselves and dosage (Figure 2). Such digital records could also serve to inform insurer and payer claims by offering accurate insight to impact on quality of life, potentially supporting decisions on dose escalations or the need to consider switching to alternate or higher efficacy medications to control symptoms and co-morbidities [20]. Although patient reported outcomes constitute components of existing diagnostic tools [19], there may be potential for higher levels of engagement if provisioned on the patient’s personal device, in an app which also imports personal images and medication logs. There may also be the potential to keep patients engaged in their care in the absence of flares or symptoms, for example, by careful design of features in the app which celebrate ‘days clear of symptoms’ through prompts. Clearly, if approached appropriately, there is potential for such an app to strengthen the interactions between patient and provider, based on longitudinally captured data and where the patient becomes more proactive in care of their condition. More specifically, given the limited time available for typical patient-physician consults, the availability of this digital data could help maximize productivity and the drive to improved outcomes.

### The Road to Implementation

The evolution of digital tools for patient self-management continues apace and in the case of CSU, the smartphone offers high potential to become a core component and contributor to patients electronic health records. In order to realize the myriad benefits and opportunities outlined herein, the following are anticipated next steps:

- Alignment between CSU patient advocacy groups and technology developers to optimize existing tools and design next generation apps and software development kits which can be customized around the individual patient
- Discussion with medical professional societies on required features, sensors, and software as medical device (SaMD) grade algorithms needed in devices and patients on which features would be of highest relevance to them
- Developing a route for integration with existing physician tools such as validated urticaria scores from which to calibrate device outputs [19]
- Technological refinement of some of the sensors and algorithms to realize medical grade precision
- Development of standardized frameworks for data capture, storage, and sharing respectful of General Data Protection Regulations
- The development of additional teledermatology media for patients (blogs, resources, apps) and the medical community (eg, scholarly journals, scientific conferences)

**Figure 3.**
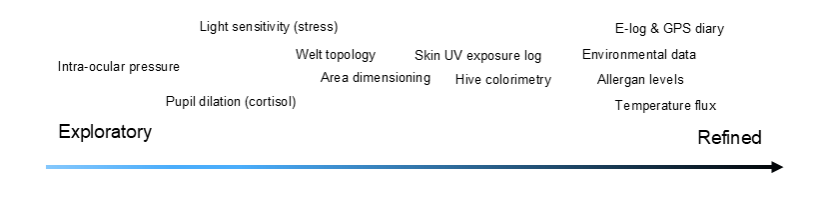
Path to maturity and validation of digital technology measures.

It is anticipated that many of the features and concepts outlined could be offered through patient support networks provisioned by developers of symptomatic and disease modifying therapies, typically through native apps or web portals. However, a key to the approach advocated herein is to more fully engage the patient on their terms, through their lived experiences in a highly personalized manner. There are two potential pathways for introduction of the features described – either as a consumer or patient support tool or through a more refined, clinical grade instrument which falls under the aegis of a regulated SaMD provisions. In either case the relative maturity of the sensors, algorithms, and their clinical validation are key considerations and there is optimism in this regard as the many features proposed are constantly evolving and refining. Although feeds on environmental data are now commonplace globally [21], and colorimetric analysis of dermatologic features is becoming more precise [22,23], many of the desired features will benefit from concerted effort for analytical refinement and clinical validation (Figure 3). Considerable progress has been made in feature dimensioning [24], and topology [8,25,26], and there is every reason to be optimistic that with collaborative efforts these can be refined for CSU applications. Likewise, metrics on ocular light sensitivity continue to evolve through measures of luminance [27,28], as do approaches to assessing pupil dilation using smartphones [29]. Even the more ambitious potential applications such as monitoring intro-ocular pressure can be accomplished with the aid of external tonometers, suggesting that future generations of devices might incorporate such functionality when miniaturized [30]. Regardless of whether such features are available in consumer products of SaMD variants, the route to implementation must also be mindful of the extant need for continual calibration of device sensors. A number of approaches to device calibration have been suggested [22,31], and systematic procedures on design of appropriate clinical trials [32]. Finally, and importantly, learnings from the ground breaking work which went in to development of existing support apps such as CRUSE can help guide adoption of appropriate controls in terms of data privacy, and provide culturally relevant learnings from the different regions where it is deployed globally [9]. In terms of clinical development, integrating learnings from the device through standardized reporting frameworks such as COSORT AI and TRIPOD AI [33,34] will allow predictive models to evolve and may help benefit related dermatological research.

The combination of smartphone sensors coupled with powerful generative AI features, both of which are trained and evolved around the individual patient themselves has a high probability of achieving the goals articulated herein and we encourage the dermatology community to embrace this vision with enthusiasm. Accordingly, we encourage active dialog in the CSU community and reader base of this journal to help realize this opportunity.

## Abbreviations

AI: artificial intelligence
CSU: Chronic spontaneous urticaria
SaMD: software as medical device

## References

[1] Zuberbier T, Abdul Latiff AH, Abuzakouk M (2022). The international EAACI/GA²LEN/EuroGuiDerm/APAAACI guideline for the definition, classification, diagnosis, and management of urticaria. Allergy.

[2] Saini SS (2014). Chronic spontaneous urticaria: etiology and pathogenesis. Immunol Allergy Clin North Am.

[3] Maurer M, Weller K, Bindslev-Jensen C (2011). Unmet clinical needs in chronic spontaneous urticaria. A GA. Allergy.

[4] Friedman A, Kwatra SG, Yosipovitch G (2024). A practical approach to diagnosing and managing chronic spontaneous urticaria. Dermatol Ther (Heidelb).

[5] Fricke J, Ávila G, Keller T (2020). Prevalence of chronic urticaria in children and adults across the globe: Systematic review with meta-analysis. Allergy.

[6] Yosipovitch G, Biazus Soares G, Mahmoud O (2023). Current and emerging therapies for chronic spontaneous urticaria: a narrative review. Dermatol Ther (Heidelb).

[7] Flores M, Glusman G, Brogaard K, Price ND, Hood L (2013). P4 medicine: how systems medicine will transform the healthcare sector and society. Per Med.

[8] Liu Y, Jain A, Eng C (2020). A deep learning system for differential diagnosis of skin diseases. Nat Med.

[9] Neisinger S, Sousa Pinto B, Ramanauskaite A (2024). CRUSE^®^ -An innovative mobile application for patient monitoring and management in chronic spontaneous urticaria. Clin Transl Allergy.

[10] Boucher A, Peters M, Jones GB (2024). How digital solutions might provide a world of new opportunities for holistic and empathic support of patients with hidradenitis suppurativa. Dermatol Ther (Heidelb).

[11] Lu Q, Schulz PJ (2024). Physician perspectives on internet-informed patients: systematic review. J Med Internet Res.

[12] Ouellette S, Rao BK (2022). Usefulness of smartphones in dermatology: a US-based review. Int J Environ Res Public Health.

[13] Zoltie T, Blome-Eberwein S, Forbes S, Theaker M, Hussain W (2022). Medical photography using mobile devices. BMJ.

[14] Saini SS, Kaplan AP (2018). Chronic spontaneous urticaria: the devil’s itch. J Allergy Clin Immunol Pract.

[15] Schwartz B, Seddon JM (1981). Increased plasma cortisol levels in ocular hypertension. Arch Ophthalmol.

[16] Langer K, Jentsch VL, Wolf OT (2022). Cortisol promotes the cognitive regulation of high intensive emotions independent of timing. Eur J Neurosci.

[17] Tomaszewska K, Słodka A, Tarkowski B, Zalewska-Janowska A (2023). Neuro-immuno-psychological aspects of chronic urticaria. J Clin Med.

[18] Salminen A, Kaarniranta K, Kauppinen A (2022). Photoaging: UV radiation-induced inflammation and immunosuppression accelerate the aging process in the skin. Inflamm Res.

[19] Weller K, Groffik A, Church MK (2014). Development and validation of the Urticaria Control Test: a patient-reported outcome instrument for assessing urticaria control. J Allergy Clin Immunol.

[20] Konstantinou GN, Podder I, Konstantinou G (2025). Mental health interventions in refractory chronic spontaneous urticaria: a call to expand treatment guidelines. Cureus.

[21] Johnston FH, Wheeler AJ, Williamson GJ (2018). Using smartphone technology to reduce health impacts from atmospheric environmental hazards. Environ Res Lett.

[22] Xuan Y, Barry C, Antipa N, Wang EJ (2023). A calibration method for smartphone camera photophlethysmography. Front Digit Health.

[23] Dugonik B, Golob M, Marhl M, Dugonik A (2025). Optimizing digital image quality for improved skin cancer detection. J Imaging.

[24] Liu C, Fan X, Guo Z, Mo Z, Chang EIC, Xu Y (2019). Wound area measurement with 3D transformation and smartphone images. BMC Bioinformatics.

[25] Nightingale RC, Ross MT, Cruz RLJ, Allenby MC, Powell SK, Woodruff MA (2021). Frugal 3D scanning using smartphones provides an accessible framework for capturing the external ear. J Plast Reconstr Aesthet Surg.

[26] Rudari M, Breuer J, Lauer H (2024). Accuracy of three-dimensional scan technology and its possible function in the field of hand surgery. Plast Reconstr Surg Glob Open.

[27] Zalewski S, Skarżyński K (2024). The photometric testing of high-resolution digital cameras from smartphones-a pilot study. Sensors (Basel).

[28] (2013). Information Visualization.

[29] Barry C, de Souza J, Xuan Y, Holden J, Granholm E, Wang EJ At-home pupillometry using smartphone facial identification cameras.

[30] Rahimi-Nasrabadi H, Moore-Stoll V, Tan J (2023). Luminance contrast shifts dominance balance between ON and OFF pathways in human vision. J Neurosci.

[31] Patonis P (2024). A comparative study on the use of smartphone cameras in photogrammetry applications. Sensors (Basel).

[32] Choi E, Long V, Phan P (2025). Protocol for a double-blinded randomised controlled trial and process evaluation of a digital psychotherapeutic app in Singapore to improve symptom burden in patients with dermatological problems. BMJ Open.

[33] Liu X, Cruz Rivera S, Moher D, Calvert MJ, Denniston AK, SPIRIT-AI and CONSORT-AI Working Group (2020). Reporting guidelines for clinical trial reports for interventions involving artificial intelligence: the CONSORT-AI extension. Nat Med.

[34] (2024). TRIPOD+AI statement: updated guidance for reporting clinical prediction models that use regression or machine learning methods. BMJ.

